# A Comparative Morphological and Anatomical Study of *Juniperus communis* L., *J. sibirica* Burgsd., and *J. pygmaea* K. Koch from Bulgaria

**DOI:** 10.3390/plants13172419

**Published:** 2024-08-29

**Authors:** Tzenka Radoukova, Ivanka Semerdjieva, Valtcho D. Zheljazkov

**Affiliations:** 1 Department of Botany and Methods of Biology Teaching, Faculty of Biology, University of Plovdiv “Paisii Hilendarski”, 24 Tzar Asen Street, 4000 Plovdiv, Bulgaria; kiprei@abv.bg; 2 Department of Botany and Agrometeorology, Agricultural University, Mendeleev 12 Street, 4000 Plovdiv, Bulgaria; v_semerdjieva@abv.bg; 3 Department of Plant and Fungal Diversity and Resources, Institute of Biodiversity and Ecosystem Research, BAS, 2, Gagarin Street, 1113 Sofia, Bulgaria; 4 Department of Crop and Soil Sciences, Oregon State University, 3050 SW Campus Way, 109 Crop Science Building, Corvallis, OR 97331, USA

**Keywords:** *Juniperus*, anatomy, morphology, systematics, SEM analysis

## Abstract

Of the six *juniper* species found in the Bulgarian flora, three of the species have controversial taxonomic positions. *Juniperus pygmaea* K. Koch and *J. sibirica* Burgsd. exhibit similar morphological characteristics to *J. communis* L. in terms of leaves and female cones (galbuli). This is one of the reasons why, in the recent taxonomic developments, *J. pygmaea* and *J. sibirica* were united in a common variety of *J. communis*, namely, *J. communis* var. *saxatilis*. However, such a grouping of species in the Flora of Bulgaria has not been adopted. This study aimed to evaluate the degrees of similarity or difference in the structure of the leaves, galbuli, seeds, and pollen of *J. communis*, *J. sibirica*, and *J. pygmaea* using the methods of comparative anatomy by light microscope (LM) and scanning electron microscopy (SEM) observations and complex morphological measurements. The working hypothesis of this study was that the three species would show a different degree of similarity with each other, which would clarify their taxonomic rank. The morphological parameters revealed differences between the length/width ratio of galbuli and seed length of the three species, while leaf characteristics (length and width) showed a stronger resemblance between *J. sibirica* and *J. pygmaea*. Furthermore, a greater distinction between the leaves and galbili of *J. communis* and *J. sibirica* was found. The SEM analyses showed variations in the seed shape and spermoderm among the three species. The shape of *J. communis* seeds was oval and elongated, while *J. pygmaea* seeds were pear-shaped, and *J. sibirica* seeds were triangular-rhombic. The length and height of striations were diverse on seed spermoderm in the three species. The epicuticular waxes of leaves, located on the tips of the anticlinal walls of the elongated epidermal cells in *J. pygmaea* and *J. communis*, were oval, while they formed raised comb-like crystals in *J. sibirica*. The morphological, anatomical, and SEM analysis affirmed the accepted taxonomic status of *J. communis* and *J. sibirica* as independent species within the Bulgarian flora. Based on most of the analyzed parameters, *J. pygmaea* exhibits significant similarity with *J. sibirica*. Additionally, the similar habitats of these two species support the determination of *J. pygmaea* as a variety or form of *J. sibirica* rather than *J. communis* (*J. sibirica* forma *pygmaea*).

## 1. Introduction

The genus *Juniperus* L. (Cupressaceae) is one of the most polymorphic genera in the order Cupressales [1]. The ongoing formative processes in the genus and high ecological plasticity are the reasons for the unstable taxonomic rank of the *Juniperus* species.

According to Yordanov [2], six species of *Juniperus* are distributed in the Flora of Bulgaria (*J. communis* L.; *J. oxycedrus* L.; *J. pygmaea* K. Koch, *J. sibirica* Burgsd; *J. excelsa* M.Bieb; and *J. sabina* L.). Two of the species, *J. pygmaea* and *J. sibirica*, have similar morphological characteristics to *J. communis* in terms of leaves and galbuli. Furthermore, in the latest taxonomic developments, this is one of the reasons why *J. pygmaea* and *J. sibirica* are united in a common variety of *J. communis*, *J. communis* var. *saxatilis* [3,4]. However, in the Flora of Bulgaria [2], the three species have been clearly distinguished as separate species. *Juniperus pygmaea* is distinguished by a smaller diameter of the galbuli; compared with *J. communis*, the leaves are uncurved, with a midvein reaching half of their length. A characteristic morphological feature of *J. sibirica* is arched, inwardly curved leaves with a broad whitish stripe on top, usually turned with their lower surfaces outward [2]. Horological data on the distribution of *J. pygmaea* in Bulgaria indicate that the species is distributed in the zone from 1500 to 1700 m asl in the regions of the mountains Stara Planina (The Balkans Mountains), Rhodopi, Slavyanka, and Belasitsa [5]. The vertical distribution of *J. sibirica* is between 1900 and 2400 m asl in Stara Planina, the Middle and Western Rhodopes, Rila, Vitosha, Pirin, and the Western Border Mountains [5]. There are also differences among *J. pygmaea*, *J. sibirica*, and *J. communis* in terms of their life forms. According to the classification of Raunkiaer [6], *J. pygmaea* and *J. sibirica* are typical nanophanerophytes (NPhs), while *J. communis*, especially at lower altitudes, appears more like a phanerophyte (Ph).

Despite a large number of publications on *Juniperus* species, including monographs [3,4], there are relatively few anatomical studies, mostly on leaves and stems [7,8,9,10,11,12,13,14]. The reviews of the *Juniperus* species have focused mostly on the stem anatomy rather than the leaf anatomy [15,16,17,18,19].

According to Köroğlu et al. [20] and Napp-Zinn [21], the following anatomical characteristics of leaves have taxonomic value in *Juniperus* species: (1) the contour of the cross-section; (2) the cover tissue (cuticle, epidermis, stomata); (3) the presence of the hypodermis below the epidermis over the entire leaf surface; (4) the features of the mesophyll, secretory canal, and median vein; and (5) the periphloem mechanical elements and of the transfusion parenchyma in the conducting bundle.

The morphological characteristics of *Juniperus* sp. are another important indicator of taxonomic value, as they allow for the establishment of intraspecific and interspecific differences. The size and degree of curvature in the leaves, the presence or absence of a central vein and its length, and the color, shape, and size of the galbuli are mainly used in the interspecific and intraspecific differentiation of juniper species [22,23,24,25,26,27]. The morphological studies on *Juniperus* sp. include metrics of leaves, pollen, seeds, etc. [28,29,30,31,32,33,34]. 

According to different authors [35,36,37], a combination of morphological and anatomical analyses is important for solving systematic cases in *Juniperus* species. Scanning electron microscopy (SEM) analyses provide additional information about the micromorphology of plants, but for *Juniperus* sp., there are relatively few SEM analyses. Analyses of the micromorphology of the epidermal complex have been demonstrated in some studies [38,39,40,41]. The surface sculpture patterns in the cuticles and stomatal complex of *Juniperus* sp. were investigated by Oladele [40]. Epicuticular waxes in *J. communis* have been studied by SEM analysis and X-ray diffraction [38]. The micromorphological features established by SEM analyses of the leaves, galbuli, and seeds of *J. drupacea*, *J. communis* L. var. *communis*, *J. communis* L. var. *saxatilis*, *J. oxycedrus* L. subsp. *oxycedrus*, and *J. oxycedrus* L. subsp. *macrocarpa*, naturally distributed in Turkey, were previously documented [20]. Until now, studies on comparative analyses of the anatomical structure, morphology, and SEM of *J. pygmaea* and *J. sibirica* have not been performed.

The aim of this study was to compare the morphological and anatomical features of *Juniperus communis*, *J. sibirica*, and *J. pygmaea*, as well as to evaluate their similarity or differences. The working hypothesis of this study was that the three species *J. communis*, *J. sibirica*, and *J. pygmaea* would show a different degree of similarity with each other, which would clarify their taxonomic rank.

## 2. Results

### 2.1. Morphological Studies

#### 2.1.1. Leaf Sizes

The comparative analysis of the mean sizes of the leaves showed a difference in the three investigated species in terms of length and the ratio of length/width (L/W) (Table 1). These differences were statistically proven. Among the studied species, *J. communis* was distinguished by the highest value for both the length (11.7 mm) and L/W (9.33 mm) of leaves. *J. sibirica* leaves had the smallest dimensions of 6.17 mm (length) and 5.25 mm (L/W) (Table 1). Overall, leaf width was a less variable parameter, and it did not have statistically proven interspecies differences (Table 1).

#### 2.1.2. Sizes of Galbuli and Seeds

According to the statistical analysis, differences in the F cones (galbuli) (length, length/width ratio) and seed length of the three species were found (Table 2). For all measured parameters (galbulus length, length/width ratio of galbuli, and seed length), the highest values were recorded for *J. sibirica* and the lowest for *J. pygmaea* (Table 2). The value for the width of the galbuli (6.23 mm) and the height of the seeds (0.23 mm) of *J. sibirica* was higher than the values for the other two species. Furthermore, the width/height ratio of *J. sibirica* seed had the lowest coefficient value (1.13) compared with the other two species. The width of the galbuli and height/width of the seeds of *J. communis* and *J. pygmaea* were similar.

*J. communis* and *J. pygmaea*, had similar galbulus widths (5.88 mm and 5.70 mm, respectively), seed widths (0.24 mm and 0.25 mm, respectively), seed heights (0.20 mm and 0.21 mm, respectively), and seed width/seed height ratios. *J. sibirica* and *J. pygmaea* had similar ratios of seed length/seed height (1.99 mm and 1.98 mm) and seed length/seed width (1.76 mm and 1.67 mm).

**Figure 1:**
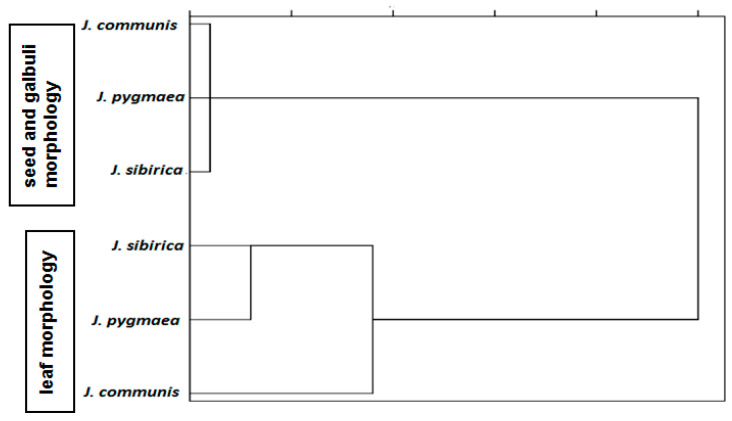
Cluster analysis of the morphological parameters of the leaves, seeds, and galbuli of *J. communs*, *J. pygmaea*, and *J. sibirica*.

The cluster analysis of the morphological parameters of the leaves, seeds, and galbuli of *J. communs*, *J. pygmaea*, and *J. sibirica* showed greater similarity between *J. sibirica* and *J. pygmaea* (Figure 1). Likewise, morphological characteristics of the galbuli and seeds showed similarity between *J. communis* and *J. pygmaea* (Figure 1).

### 2.2. Scanning Electron Microscopy (SEM) Analysis of Seeds, Leaves, and Pollen 

#### 2.2.1. Seed Characters 

##### Seed Shape 

**Figure 2:**
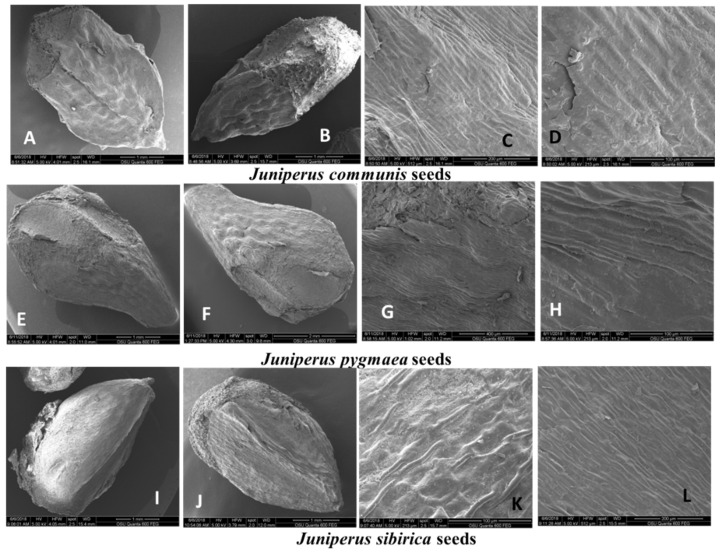
The seed form and surfaces of *Juniperus communis* (**A**–**D**), *J. pygmaea* (**E**–**H**), and *J. sibirica* (**I**–**L**). *J. communis*: (**A**)—General view of seeds, oval-elongated form and elongated conical tip; (**B**)—lateral view, elongated conical tip; (**C**,**D**)—spermoderm, anticlinal walls; *J. pygmaea*; (**E**)—general view of seeds, oval-rounded base; (**F**)—oval-elongate forms; (**G**,**H**)—the edge of the anticlinal walls; *J. sibirica*: (**J**,**F**)—triangular-rhombic, rounded base; and (**G**,**H**)—spermoderm with smooth anticlinal cell walls with an oval edge.

The shape of the seeds in the three species varied from oval-elongated (*J. communis*) to pear-shaped (*J. pygmaea*) and triangular-rhombic (*J. sibirica*). The seeds of *J. communis* had a rounded base and a flattened, elongated conical tip (Figure 2A–D). The seeds of *J. sibirica* were triangular-rhombic, with a rounded base, from which edges started along the entire seed length. The apex of the seeds in this species was bluntly conical (Figure 2I–L). The seeds of *J. pygmaea* were oval-elongate, and pear-shaped, with an oval-rounded base and elongated oval apex (Figure 2E–H). Striations varying in length and height were observed on the surface of the seeds of the three species.

##### Seed Spermoderm

**Figure 3:**
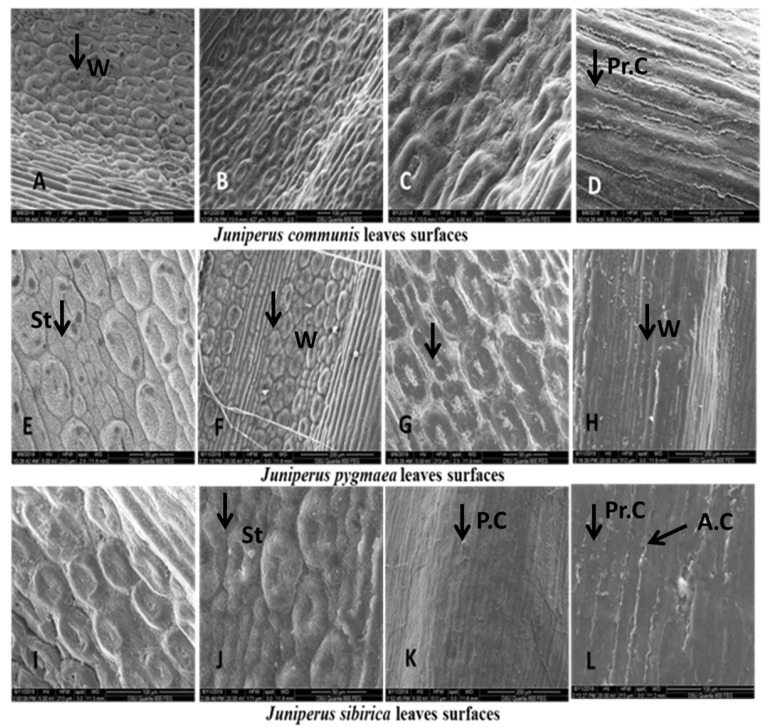
The leaf surfaces of *Juniperus communis* (**A**–**D**), *J. pygmaea* (**E**–**H**), and *J. sibirica* (**I**–**L**). The shape of the prosenchyma form of epidermal cells (Pr.Cs) (**D**,**L**), oval forms of anticlinal cells (A.Cs) (**L**); smooth periclinal cell walls (P.C); and waxes (Ws) (**A**,**H**,**F**).

**Figure 4:**
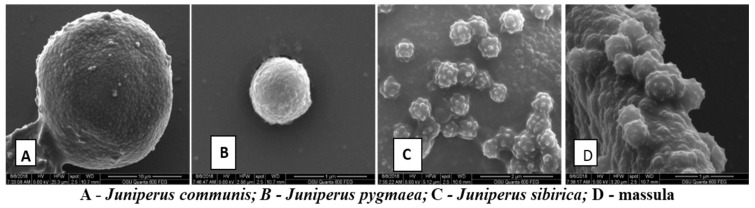
The general view of the pollen of *Juniperus communis* (**A**), *J. pygmaea* (**B**), and *J. sibirica* (**C**) and (**D**) the massula of *J. sibirica*.

According to Barthlott and Ehler [42], the seed surface could be described as Tabular-type to Convex-type in three species (Figure 2). The anticlinal walls were unevenly convex. The anticlinal cell wall boundaries were smooth with an oval edge in *J. communis* and *J. sibirica*. In *J. pygmaea*, the edge of the anticlinal walls was pointed (Figure 2, Figure 3 and Figure 4). The anticlinal cell walls are generally well-developed. The periclinal cell walls in all three studied species were smooth with slight striations on them (Figure 2).

#### 2.2.2. Leaf Surfaces

##### Epicuticular Waxes

Massive wax production formed a very dense layer of membranous platelets on the epidermal and guard cells in the stomata of the three species. In separate areas on the surface of the epidermis, many platelets appear as crusts (Figure 3A–L). The epicuticular waxes, located on the tips of the anticlinal walls of the elongated epidermal cells in *J. pygmaea* and *J. communis*, were oval, while they formed raised comb-like crystals in *J. sibirica* (Figure 3A–L).

##### Epidermal Cells

The shape of epidermal cells was elongated in the prosenchyma form in the three species. The anticlinal cell wall boundaries were oval forms. The periclinal cell walls were smooth.

#### 2.2.3. Pollen Morphology

Overall, in this study, the pollen grains of *J. communis*, *J. sibirica*, and *J. pygmaea* were oblate spheroidal and monoporate with circular pores. The sexine of the pollen surface was scabrate–granulose, according to Punt et al. [43]. The pollen grains of three species were isolated, or, in some instances, they were either in larger or smaller groups of massula (Figure 4A–D). 

### 2.3. Light Microscopic Analysis of the Internal Structure of Leaves

#### 2.3.1. Shape of Leaf Cross-Sections in *J. communis*, *J. sibirica*, and *J. pygmaea*

**Figure 5:**
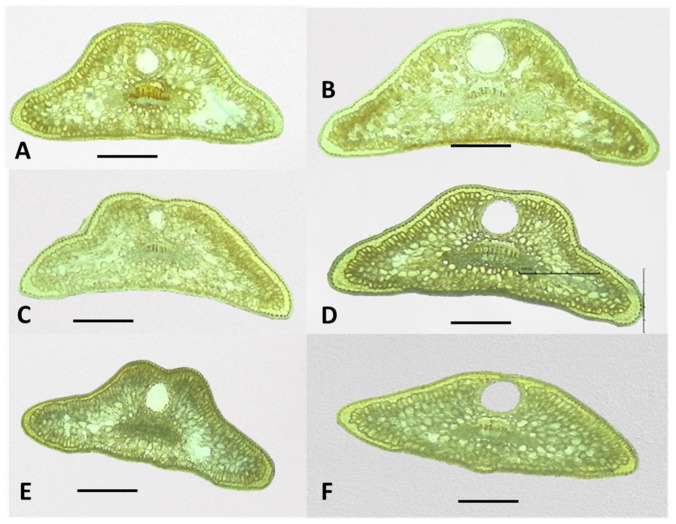
The general view of the leaf shapes of *Juniperus communis*: Population Bekleme, (**A**)—male, (**B**)—female; population Dobrostan, (**C**)—male, (**D**)—female; and population Markovo, (**E**)—male, (**F**)—female. The photos were taken with LM Motic DMA, (×4); scale bar = 100 µm.

**Figure 6:**
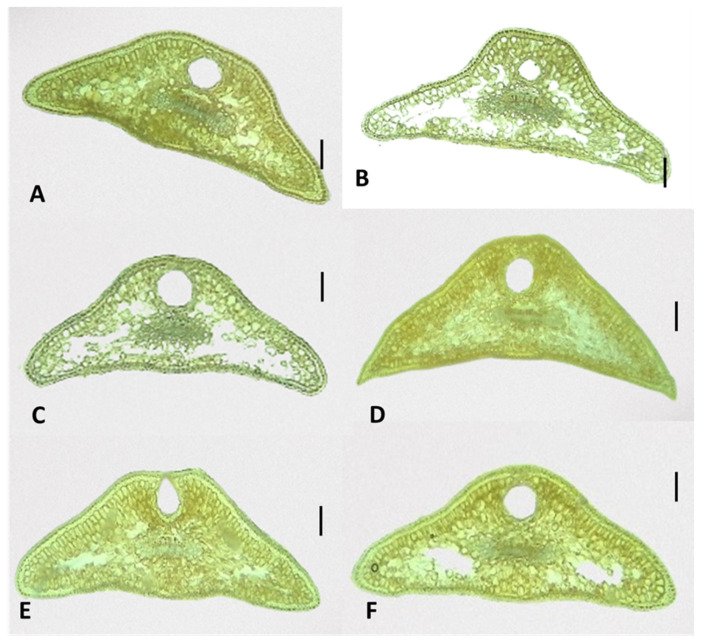
The general view of the leaf shapes of *Juniperus sibirica*: population Bekleme, (**A**)—male, (**B**)—female; population Pirin, (**C**)—male, (**D**)—female; and population Vitosha, (**E**)—male, (**F**)—female. The photos were taken with LM Motic DMA, (×4); scale bar = 100 µm.

**Figure 7:**
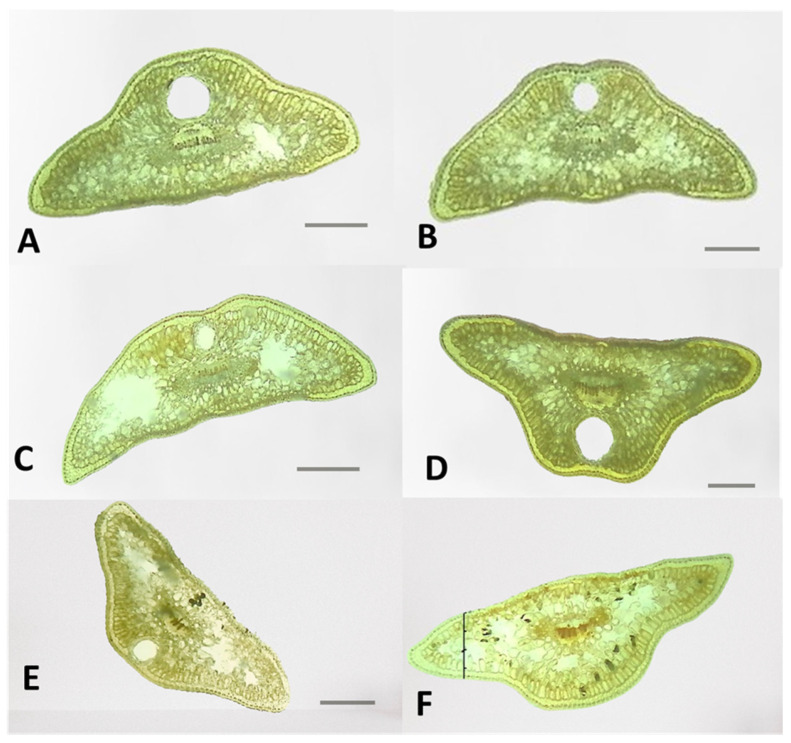
The general view of the leaf shapes of *Juniperus pygmaea*: population Kamenlivitsa, (**A**)—male, (**B**)—female; population Dobrostan, (**C**)—male, (**D**)—female; and population Mursalitsa, (**E**)—male, (**F**)—female. The photos were taken with LM Motic DMA, (×4); scale bar = 100 µm.

The cross-sections of the leaves of the three *Juniperus* species showed that the basic shape of the leaves was trapezoidal (Figure 5, Figure 6 and Figure 7). Deviations from the basic trapezoidal shape were observed, both among the three juniper species and in some populations of the same species. 

The comparative analysis of the coefficient characterizing the shape of the leaves (CC) did not show a clear differentiation among the three studied species. For example, the female plants of *J. communis* from Markovo had the highest mean CC coefficient value (1.32), while the male plants of *J. sibirica* from Pirin (0.9) had the lowest (Table 3).

The leaf height (LH) was highest for the female plants of *J. communis* from Bekleme (666.4 µm); however, the female plants of *J. communis* from Markovo had the lowest value (390.6 µm) (Table 3).

#### 2.3.2. Anatomical Structure of the Leaves of *J. communis*, *J. sibirica*, and *J. pygmaea* by the LM

**Figure 8:**
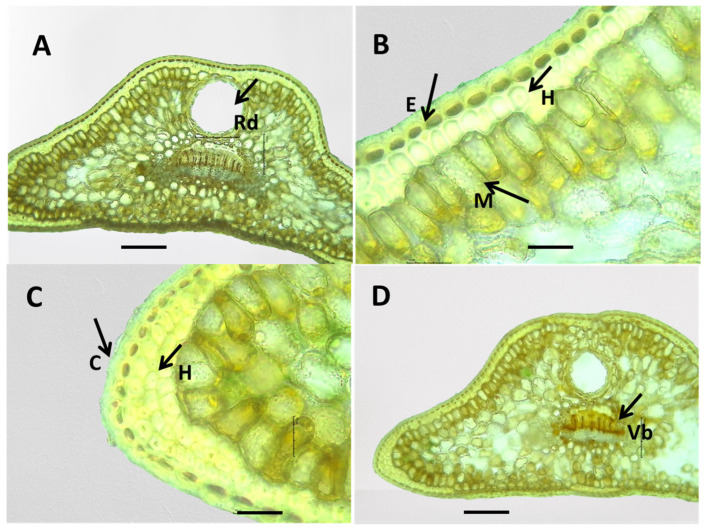
Anatomical features of the internal structure of *Juniperus communis* leaves. Resin duct (Rd)—(**A**). Epidermis (E), hypodermis (H), mesophyll (M)—(**B**). Cuticle (C), hypodermis (H)—(**C**). Vascular bundle (Vb)—(**D**). The photos were taken with the LM Motic DMA; (×10—(**A**,**D**)) (×40—(**B**,**C**)); scale bar = 100 µm.

**Figure 9:**
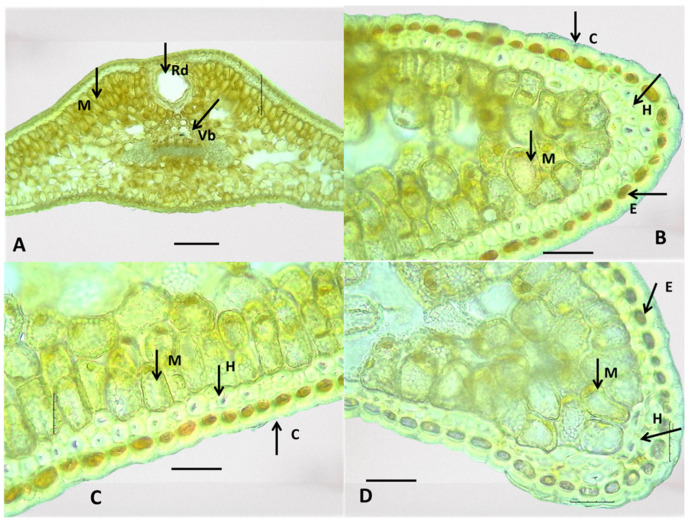
Anatomical features of the internal structure of *Juniperus sibirica* leaves. Resin duct (Rd), mesophyll (M), vascular bundle (Vb)—(**A**). Epidermis (E), hypodermis (H), mesophyll (M), cuticle (C)—(**B**). Hypodermis (H), mesophyll (M), cuticle (C)—(**C**). Epidermis (E), mesophyll (M), hypodermis (H)—(**D**). The photos were taken with the LM Motic DMA; (×10—(**A**)) (×40—(**B**–**D**)); scale bar = 100 µm.

**Figure 10:**
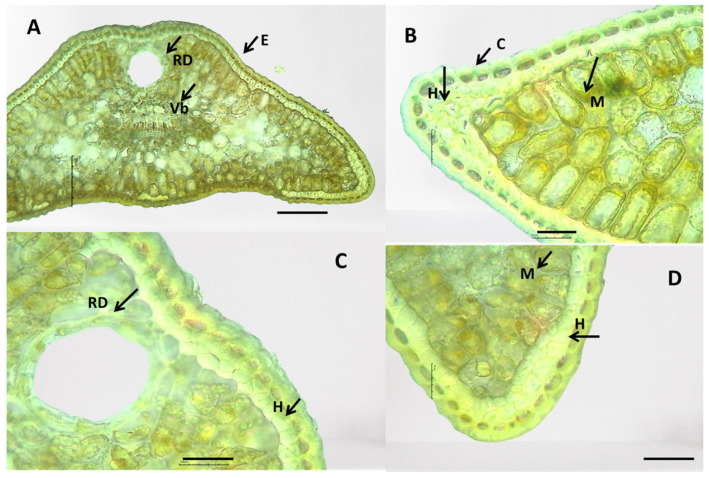
Anatomical features of the internal structure of *Juniperus pygmaea* leaves. Resin duct (Rd), vascular bundle (Vb), epidermis (E)—(**A**). Hypodermis (H), mesophyll (M), cuticle (C)—(**B**). Hypodermis (H), resin duct (Rd)—(**C**). Mesophyll (M), hypodermis (H)—(**D**). The photos were taken with the LM Motic DMA; (×10—(**A**,**D**)) (×40—(**B**,**C**)); scale bar = 100 µm.

The anatomical stricture of the leaves in the three studied *Juniperus* species showed similarity. The results are presented in Table 4 and Figure 3 and Figure 8, Figure 9 and Figure 10.

Epidermis (E): The covering tissue of the leaves of the three juniper species was present as a single layer and cuticle. The cuticle was thick and penetrated among the cells (Figure 8, Figure 9 and Figure 10). The main epidermal cells of the epidermis were elongated, rectangular in shape (prosenchyma form), and thick-walled (Figure 3). In the cross-section, the epidermal cells had a rounded cubical shape with a convex outer cell wall and small lumens (Figure 8, Figure 9 and Figure 10). The leaves of all three *Juniperus* species were epistomatic. The stomata were below the level of the epidermis and had formed sub-stomatal cavities (Figure 3). The stomata were surrounded by four to six peristomatal cells, two of which were located parallel and two perpendicular to the closing cells (Figure 3).

Hypodermis (H): The hypodermis of the three *Juniperus* species was single-layered beneath the epidermis and interrupted below the stomata. A continuous upper hypodermis was observed mostly in the middle part of the leaves in all three examined species (Figure 8, Figure 9 and Figure 10). The hypodermis was one or two-layered to multi-layered in the corners of the leaves (Figure 8, Figure 9 and Figure 10).

Mesophyll (M): The mesophyll of the leaves of the three *Juniperus* species was composed of spongy and palisade parenchyma cells (Figure 8, Figure 9 and Figure 10). The palisade parenchyma consisted of two to three layers of closely spaced cylindrical cells (Figure 8B, Figure 9B, Figure 10B,D). The spongy parenchyma consisted of oval cells with more or less pronounced intercellular spaces (Figure 8, Figure 9 and Figure 10).

Conductive system: The conduction system of the three examined *Juniperus* species was represented by one collateral vascular bundle (Vb) located in the central part of the leaf and forming a central vein (Figure 8, Figure 9 and Figure 10). The transfused tissue was enveloped by a clearly differentiated endoderm, which was better developed towards the corners of the leaves. The xylem was represented by tracheids located towards the upper side of the leaf. The phloem was made up of lattice cells towards the underside of the leaf. A layer of sclerenchyma cells was underneath the phloem (Figure 8, Figure 9 and Figure 10).

Resin duct (Rd): In all examined *Juniperus* species, a large rounded schizogen resin duct was located under the conducting bundle in the central part of the leaf (Figure 8, Figure 9 and Figure 10). The secretory duct was separated from the mesophyll by a layer of cells with thickened cell walls having a mechanical function. On the inner side of the mechanic cells was a row of thin-walled secretory cells. The dimensions of the resin canal were variable among species and populations of the same species. 

The statistical differences with the highest values for *J. communis* were as follows: leaf height (LH) (545.7 µm), the thickness of the upper cover tissue (TUCT) (7.1 µm), the thickness of the hypodermis lower surface (THLS) (5.4 µm), the thickness of the hypodermis leaf angles (THLAs) (10.9 µm), the width of the median vein (WMV) (435.6 µm), the width of the aperture of the secretory canal (WASC) (152.4 µm), the height of the aperture of the secretory canal (HASC) (167.2 µm), stomata number (SN) (1529.7 µm), stomata width (SW) (5.9 µm), and stomata length (SL) (9.8 µm) (Table 4).

In *J. sibirica*, the statistically highest values were the thickness of the lower cover tissue (TLCT)—5.1 µm, the thickness of the palisade parenchyma (TPP)—95.1 µm, the thickness of the hypodermis upper surface (THUS)—3.1 µm, the thickness of the hypodermis lower surface (THLS)—3.8 µm, the thickness of the spongy parenchyma (TSP)—186.2 µm, and the height of the median vein (HMV)—131.4 µm. The stomata number was the lowest (SN)—1005.7. The values of the other indicators were not statistically different (Table 4).

The measured parameters of the *J. pygmaea* leaves (i.e., the leaf height (LH), the thickness of the lower cover tissue (TLCT), the width of the aperture of the secretory canal (WASC), the height of the aperture of the secretory canal (HASC), and stomata width (SW)) were lower than those of *J. communis* and *J. sibirica*. Of the analyzed histological structures of *J.pygmaea* leaves, the hypodermis was an exception, where the upper surface thickness (TUCT) had the highest average value of 7.1 µm.

**Figure 11:**
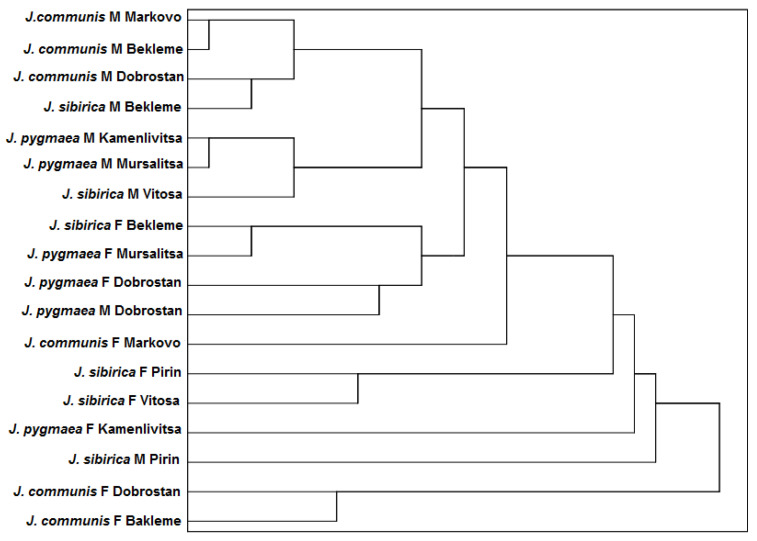
Cluster analysis regarding the anatomical parameters of the leaves of the three studied species including the populations and sex of the plants.

The cluster analysis of the studied anatomical parameters revealed clear groupings according to gender. All male plants of *J. communis* were united in an independent cluster, in which the male plants of *J. sibirica* from population Bekleme were also included. Similarly, the male plants of *J. pygmaea* from populations Kamenlivitsa and Mursalitsa were grouped into a separate cluster, along with the males of *J. sibirica* from population Vitosa. The female plants of *J. sibirica* from the population Troyan and *J. pygmaea* from Mursalitsa formed a distinct cluster, which also included the female plants of *J. pygmaea* from the population Dobrostan. A separate cluster, though at a greater distance from the aforementioned group, included the female plants of *J. sibirica* from Pirin and Vitosa. The remaining variants showed a higher degree of separation, with the most significant differences observed in the female plants of *J. communis* from Bekleme and Dobrostan (Figure 11).

**Figure 12:**
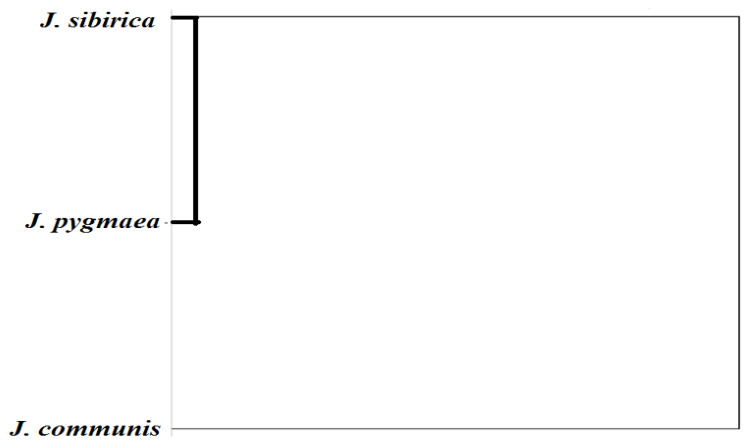
Cluster analysis of the studied anatomical parameters at the species level.

The cluster analysis of the studied anatomical parameters showed a similarity between *J. sibirica* and *J. pygmaea* and a greater distance between *J. sibirica* and *J. communis* (Figure 12). 

## 3. Discussion

### 3.1. Morphological Studies

Variability in leaf shape is closely related to physiological potential and to genetic differences among species [3]. Parameters such as leaf width and length, degree of curvature, and the width/length ratio are important when studying the systematics and ecology of junipers [1]. In the present study, leaf length was the most variable parameter. Despite the greatest ecological plasticity in the leaves of *J. communis* reported by a number of authors [1,4,44,45], in our study, the strongest variation in leaf length was found in *J. pygmaea*.

The studies by Brus et al. [28], Adams [46], Mazur et al. [32], and Ward [47] on different species of the juniper genus showed a different degree (high–low) of intra-species and intra-population morphological variability. Some authors believe that this is not related to geographic, altitude, or other environmental variables [48], but, according to others, it is mainly due to these factors [32,46]. The specific evolutionary history of each species plays an important role in determining the distribution level of its genetic diversity and the degree of variation in its morphological–anatomical features [49].

In this study, the length of the leaves and the length-to-width ratio were the most objective morphological parameters for distinguishing *J. communis*, *J. sibirica*, and *J. pygmaea*. For both parameters, *J. communis* exhibited the maximum values, while *J. sibirica* showed the minimum values. The results of this study align with those of Vasić and Dubak [12], who reported similar findings in their comparative analysis of the leaf length, width, and thickness of *J. sibirica* and *J. communis*. The latter authors demonstrated that *J. sibirica* had significantly shorter, narrower, and thinner leaves. According to Vasić and Dubak [12], the differences in leaf length between the two species were statistically significant, while the differences in width and thickness were minimal. Our study confirmed these findings.

Overall, the results from this study regarding leaf length are consistent with the data presented in the Flora of Bulgaria [2]. *Juniperus communis* stands out with the longest leaves (10–15 mm, according to the Flora of Bulgaria; 10.65–13.44 mm, according to our study). *Juniperus sibirica* had the shortest leaves (4–8 mm, according to the Flora of Bulgaria; 5.27–7.85 mm, according to our data). *Juniperus pygmaea* had an intermediate leaf length (6–10 mm, according to the Flora of Bulgaria; 5.94–11.21 mm, according to our data). Knyazeva [45] also noted similar differences in the length of the leaves of *J. communis* and *J. sibirica*. According to the data provided by the latter author, the leaves of *J. sibirica* were needle-like, narrow-lanceolate, prickly, and 4–8 (10) mm long, while the leaves of *J. communis* were elongate-pointed, grooved at the top, linear, and 4–15 mm long.

One of the most important characteristics of the genus *Juniperus* is the morphological features of the female cones (galbuli). They are formed in 12 to 24 (36) months, contain from 1 to 12 seeds (depending on the species), and have a hard seed coat [50,51,52,53,54,55]. The fleshy juicy (berry-like) shell of the galbula is formed as a result of the growth of the seed coats [1,33,51]. The anatomy of galbuli and seeds in the genus *Juniperus* is similar, but the shape, size, and color of the galbuli are some of the main taxonomic characteristics that distinguish species [56]. The morphology of the galbuli was the basis for the taxonomic classification of the genus *Juniperus* and the determination of sect. *Juniperus* and section *Sabina* [33]. As mentioned in the Introduction, *J. communis*, *J. pygmaea*, and *J. sibirica* belong to the sect. *Juniperus*, and they have the same color and structure of the galbuli. The search for species differences is based on the metric values of the investigated morphological indicators. One of the most commonly used parameters in the taxonomy of juniper species is the size of mature galbuli and seeds [57]. Despite the taxonomic value of this feature, it is often a prerequisite for determining a large number of subspecies, forms, races, geographical varieties, and climatic ecotypes in the genus, which further complicates the taxonomy of the species [58].

According to this study, the length of the galbuli and seeds, as well as the ratio (L/W) of the galbuli were significantly different among *J. communis*, *J. sibirica*, and *J. pygmaea*. The statistically significant differences in the sizes of the galbuli and seeds of the three species determined in this study have some similarities with those indicated in the Flora of Bulgaria [2].

### 3.2. Scanning Electron Microscopy (SEM) Analysis of Seeds, Leaves, and Pollen 

#### 3.2.1. Seed Characters

As stated, galbuli (F cones) are used as the main taxonomic character to distinguish the species of the genus *Juniperus*. Research interest in juniper seeds has most often focused on their size, as seed size is related to seed dispersal and germination [59]. Despite the intensive study of the genus, comparative analyses of the microstructure of the spermoderm of *J. communis*, *J. sibirica*, and *J. pygmaea* seeds are scarce. One of the possible reasons is the unclear taxonomic status of *J. sibirica* and *J. pygmaea.* According to Kӧroğlu et al. [20], the spermoderm of *J. communis* from Turkey is striate-reticulate. The results of this study indicated that the spermoderm of all three species (*J. communis*, *J. sibirica*, and *J. pygmaea*) ranged from Tabular-type to Convex-type. The seeds of *J. communis* and *J. sibirica* were relatively similar in shape, while the seeds of *J. pygmaea* exhibited distinct differences.

#### 3.2.2. Leaf Surfaces

The microstructure of epicuticular waxes and the epidermal surface of the leaf is an important key in plant taxonomy [60]. Epicuticular waxes and cuticles provide additional information about species, but they are influenced by a number of factors, such as environmental conditions, plant organs, and ontogenetic development [61]. In this study, the shape of the epidermal cells and the layer of epicuticular waxes were found to be similar among the three species. The only observed difference in this study was the raised ridge-like crystals of the epicuticular waxes on the edge of the incline walls in *J. sibirica*. This was probably the result of the extreme climatic conditions under which the species developed.

#### 3.2.3. Pollen

Generally, the characteristics of pollen and its sexine are an important indicator in plant taxonomy, especially among different plant genera and families. Peculiarities of sexine (the outer layer of the exine), ornamentation, and the presence or absence of pores, colpi, and mura can provide important evidence for evolutionary relationships in plant systematics [62]. In this study, the SEM analyses revealed that the pollen of the three species (*J. communis*, *J. sibirica*, and *J. pygmaea*) was similar. This result was expected because the reproductive system of plants is quite conservative [63]. Furthermore, as demonstrated by Halbritter et al. [64], when the pollen of a taxon (representing a family or genus) is similar among species, it is termed stenopalynous-type. Therefore, the pollen grains in the three species are likely stenopalynous-type.

### 3.3. Anatomical Studies

The analysis of anatomical characteristics, particularly the shape of the leaves, identified leaf height (LH) as the most important feature for distinguishing the three species, with statistically significant differences among them. In this study, a maximum value for the indicator (LH) was found for *J. communis*, and a minimum was found for *J. pygmaea*. Similar to this study, was the study by Lakušic and Lakušic [8] whichfound no statistically significant differences in leaf width (LW) or leaf thickness (LT) between *J. communis* and *J. alpina* [syn. *J. sibirca*]. The width and thickness of the leaves of *Juniperus* species may vary with environmental conditions, altitude, and the degree of available moisture. For example, for *J. communis*, Vasič et al. [65] found lower LT values at lower altitudes. According to Mikheeva [44], a key characteristic of the leaf shape in *Juniperus* is the CC index (coefficient of curvature of the leaves in cross-section). Our study found a value of 0.99, which is similar to the value reported by Mikheeva [44] for *J. communis*.

Vasič et al. [65] noted that there were no significant differences in the thickness of the upper and lower epidermis in *J. communis*. Our study identified the thickness of the hypodermis on the lower surface (TLCT) as a statistically significant indicator among the three studied species, with *J. sibirica* showing the maximum value and *J. pygmaea* showing the minimum value. A similar statistically significant difference in the thickness of the upper and lower epidermis between *J. communis* and *J. alpina* [syn. *J. sibirca*] was also listed by Vasič and Dubak [12].

According to Ivanescu et al. [66], the leaves of *J. communis* were amphistomatic, but in this study, we observed stomata only on the upper epidermis, so the leaves were epistomatic. The results from this study align with Knyazeva’s [45] findings for the leaves of *J. communis* var. *communis* and *J. communis* var. *saxatilis* (syn. *J. sibirica*). In this study, we identified statistically significant differences in the stomata number (SN) and stomata length (SL), with *J. communis* exhibiting the highest values for both indicators. According to Vasič and Dubak [12], changes in the thickness of the epidermis is often due to moisture deficiency, which leads to a decrease in both thickness and the number of stomata. Their research indicates a statistically significant lower number of stomata in *J. sibirica* compared with *J. communis*, a finding corroborated by our study.

According to Güvenç et al. [17], the hypodermis is absent in the middle part of the upper surface of *J. communis* var. *communis* leaves. However, in the *Juniperus* species examined in this study, the hypodermis was present on both sides of the leaves. Furthermore, this study found that in the middle part, the cells of the hypodermis were smaller in size than the cells of the angles, and its walls were not strongly lignified.

The thickness of the hypodermis on the upper surface (THUS) and the lower surface (THLS) showed significant differences among the three species, with *J. communis* exhibiting the greatest thickness and *J. sibirica* showing the least. According to Mikheeva [44], the thickness of the mechanical tissue in *Juniperus* is greater in dry habitats, which is most likely related to an increase in the size of the cells or the thickness of their walls.

Overall, in this study, the anatomical structure of the leaves of the three studied species (*J. communis*, *J. sibirica*, and *J. pygmaea*) showed similar histological characteristics. Differences were observed in the thickness of the individual layers. For example, the thickness of the hypodermis in the leaf angles varied among the three species. Also, variations were found in the thickness of the palisade parenchyma (TPP) and the thickness of the spongy parenchyma (TSP). The most pronounced variation was found in *J. sibirica*, which exhibited a maximum TPP and a minimum TSP.

The significant variation in the two parameters was attributed to the different number of palisade parenchyma layers, either one or two. Güvenç et al. [17] reported that the palisade parenchyma in *J. communis* var. *communis* and *J. communis* var. *saxatilis* (syn. *J. sibirica*) can be either single-layered or double-layered. According to Lakušić and Lakušić [8], the mesophyll in *J. communis* ssp. *communis* var. *communis* and *J. communis* ssp. *alpina* (syn. *J. sibirica*) consists of one layer of palisade cells on the adaxial side and one to two layers on the abaxial side. Mikheeva [44] noted that the parenchyma area in juniper leaves increases in habitats with sufficient moisture. Conversely, a reduction in parenchyma area is associated with a greater thickness of covering tissues, as well as the area of the resin canal and conducting bundle, with the mesophyll occupying 67–72% of the total leaf cross-sectional area in *Juniperus* species.

According to Vasič and Dubak [12], a key difference between *J. communis* and *J. sibirica* lies in the size of the conducting bundle, which is significantly smaller in *J. sibirica*. However, in our study, the size of the conducting bundle did not provide a clear distinction between the three species. While *J. communis* had the maximum width of the median vein (WMV), the height of the median vein (HMV) showed no significant difference between *J. communis* and *J. pygmaea*. Mikheeva [44] noted that habitat conditions significantly influence the area of the conduction system in juniper leaves. She reported that in environments with abundant moisture, the cross-sectional area of the vein increases by 4–9%. Similarly, Vasič et al. [65] observed that the dimensions of the central vein in various *Juniperus* species of the section *Juniperus* tend to increase at higher altitudes. According to Serebryanaya and Karpenko [9], the position and dimensions of the resin canal are among the most important taxonomic characteristics of the genus *Juniperus*. Our research supports this, as we found statistically significant differences in the width (WASC) and height (HASC) of the aperture of the secretory canal among the three studied species. *J. communis* exhibited the highest values for both parameters, while *J. pygmaea* had the lowest.

According to Vasič and Dubak [12], the size difference in the resin duct between *J. communis* and *J. sibirica* is not significant, though *J. communis* tended to have slightly larger ducts. Lakušic and Lakušic [8] reported larger resin canals in *J. communis* ssp. *alpina* (syn. *J. sibirica*), but this difference in *J. communis* ssp. *communis* var. *communis* was also considered non-essential. Mikheeva [44] noted that the size of the resin duct in junipers varies with the age of the leaves and habitat conditions.

An interesting finding of our study was the relationship between resin duct sizes and the sexual differentiation of the three *Juniperus* species. Most female plants had larger resin ducts, and this distinction, along with the clear grouping of most male plants in the cluster analysis, suggests that sexual dimorphism is a significant factor influencing the anatomical parameters of junipers. Knyazeva [1] also found reliably larger leaf sizes in female individuals of *J. communis* from high mountain habitats. However, according to Knyazeva [1], the degree of variability in the vegetative and generative organs of junipers depends more on the characteristics of the specific trait than on sex.

## 4. Materials and Methods

### 4.1. Materials

Materials (leaves of M and F plants, F cones (galbuli), pollen of M plants) from *J. sibirica*, *J. pygmaea*, and *J. communis* were collected from three populations in Bulgaria, as shown in Table S1 and Figure S1. The populations are from different floristic regions, and they are at a relatively large geographical distance. The exact GPS coordinates and altitude of the studied populations are presented in Table S1. The collected material was deposited in the herbarium of Agricultural University, Plovdiv (SOA). The voucher numbers of the herbarium specimens are 059854-059869.

### 4.2. Methods

#### 4.2.1. Morphological Studies

Morphological studies of *J. communis*, *J. sibirica*, and *J. pygmaea* were carried out on fresh leaves and galbuli in laboratory conditions. Study materials were sampled from well-developed plants, from the middle part of two-year-old twigs. The leaves and galbuli were collected from sunny, south-facing parts of the sampled twigs. The following morphological biometrics were studied: (1) leaves—length and width in mm; (2) female cones (galbuli)—length (from the grip area on the twig to the base of the galbula) and width (equatorial diameter) in mm; and (3) seeds—number, length, width, and height, to the nearest 0.01 mm. The morphological features of the leaves, and the gender of the plants (male, female) were taken into account. This research was carried out according to the methodology of Ermolina [41], Klimko et al. [30], and Knyazeva [31]. For each indicator, 50 measurements were taken from each population.

#### 4.2.2. Scanning Electron Microscopy (SEM) Analysis of Leaves, Seeds, and Pollen Grains

The scanning electron microscope (SEM) used in this investigation was an FEI Quanta 600 SEM at the Microscopy Facility at Oregon State University, United States. Sample preparation included placing small samples into a fixative of 1% paraformaldehyde and 2.5% glutaraldehyde in 0.1M sodium cacodylate buffer with a pH of 7.4. The samples were soaked in the fixative for 2 h, followed by two rinses in 0.1M Cacodyalte buffer, 15 min each, and dehydration in acetone (10%, 30, 50, 70, 90, 95, 100%), 10–15 min each, followed by critical point drying (two “bomb flushes” at chamber pressure to 5 °C, chamber filled with CO_2_). The samples were left to vent for 5 min, and then, the procedure was repeated. The dry samples were mounted onto an aluminum SEM stub with double-stick carbon tape. The samples were sputter-coated with a Cressington 108A sputter coater from Ted Pella with Au/Pd, 60/40 mix. 

For leaf surfaces, the terminology and classification of Barthlott et al. [60] were used.

For seed morphology descriptions of species, the shapes, as well as the structure of the spermoderm, were determined. In this case, the terminology and classification described by Barthlott and Ehler [42] were used. For pollen surfaces, we used the terminology and classification described by Punt et al. [43].

#### 4.2.3. Anatomical Studies

Anatomical studies were performed on fully developed leaves of the target species from the populations indicated in Table S2. The leaves were fixed in 70% ethanol. Semi-permanent microscopic preparations were prepared from the middle part of the leaf blade. The studied anatomical indicators are presented in Table S2 and Figure S2. For each indicator, 30 measurements were made. Observations were performed with a light microscope Magnum T. The photos were taken with LM Motic DMA, with the documentation system Moticam A5, 5MP live resolution, in the laboratory of the Department of Botany and Agrometeorology at Agricultural University, Plovdiv. To characterize the shape of the leaves according to the methodology of Mikheeva [44], the coefficient of curvature (CC) of the cross-section of the leaves was calculated according to the formula:

CC = t/h,

where (t) is the leaf width and (h) is the leaf height. The coefficient characterizes the degree of curvature of the adaxial side (ad) of juniper leaves

#### 4.2.4. Statistical Methods

The statistical packages SPSS and Statistics were used to assess statistically significant differences in the quantitative markers. 

When determining the interspecies, gender, and population differences in the metric indicators, the Duncan multiple range test was used, taking into account the statistically significant differences in the arithmetic means. 

A Hierarchical Cluster Analysis was used to compare the relationship between the indicators (between groups linkage) using the method of the squared Euclidian distance (squared Euclidian distance). Clusters were formed showing the similarities in the measured indicators at the interspecies, intersex, and population levels.

## 5. Conclusions

The length of the leaves and the ratio between the length and width of the leaves were significant indicators in the taxonomic differentiation of *J. communis*, *J. sibirica*, and *J. pygmaea*. The leaves of *J. communis* had maximum values, while those of *J. sibirica* had minimum values. 

Overall, the length, L/W ratio of F cones, and seed length are reliable taxonomic marks for the differentiation of *J. communis*, *J. sibirica*, and *J. pygmaea*, with the highest values for *J. sibirica* and the lowest for *J. pygmaea*.

Morphological analysis of the leaves revealed similarities between *J. sibirica* and *J. pygmaea*, while similarities in galbuli and seeds were observed between *J. communis* and *J. pygmaea*. A greater distance in both cases was observed between *J. communis* and *J. sibirica*.

Anatomical analysis revealed differences among the three species in leaf height, stomata (size and number), and resin canal dimensions. In all these parameters, *J. communis* exhibited the highest values.

The anatomical similarities among the three species mirrored those observed in their leaf morphology, with a stronger resemblance between *J. sibirica* and *J. pygmaea*, while *J. communis* showed greater divergence from *J. sibirica*.

The morphological and anatomical analysis (LM, SEM) of both vegetative and generative organs in the three studied species supports the classification of *J. communis* and *J. sibirica* as distinct species within the Bulgarian flora. However, it raises questions about the taxonomic position of *J. pygmaea*. Based on the majority of the examined traits, *J. pygmaea* shows a closer resemblance to *J. sibirica*. Coupled with their shared habitats, this suggests that *J. pygmaea* may be more accurately classified as a variety or form of the Siberian juniper, *J. sibirica* forma *pygmaea*, rather than as a distinct species.

## Figures and Tables

**Table 1:** Determined average leaf length and width (mm) and the leaf L/W ratio for the three studied species. Arithmetic means marked with the same letters have no statistically proven differences.

Species	Leaf Length *	Leaf Width	Leaf L/W Ratio
*J. communis*	11.7 a	1.30 a	9.33 a
*J. sibirica*	6.17 c	1.26 a	5.25 c
*J. pygmaea*	8.18 b	1.19 b	7.50 b

* For each species, 50 individual leaves per population were measured.

**Table 2:** The mean galbulus length; width; number of seeds; and seed length, width, and height for the galbuli and seeds (mm) of the three studied species.

Species	Galbuli Length	Galbuli Width	L/W Galbuli	Number of Seeds	Seed Length	Seed Width	L/W Seeds	Seed Height	L/H Seeds	W/H Seeds
*J. communis*	6.00 b*	5.88 b	1.04 b	3 a	0.42 b	0.24 b	1.87 a	0.20 b	2.21 a	1.20 a
*J. sibirica*	6.65 a	6.23 a	1.07 a	2.9 a	0.46 a	0.26 a	1.76 b	0.23 a	1.99 b	1.13 b
*J. pygmaea*	5.56 c	5.70 b	0.98 c	3 a	0.41 c	0.25 a,b	1.67 b	0.21 b	1.98 b	1.20 a

* Arithmetic means marked with the same letters have no statistically proven differences.

**Table 3:** Duncan tests of the mean values in relation to the shape of the leaves of the studied variants of the three *Juniperus* species.

Population	Sex	Index	
		LH	CC
*J. communis*			
Dobrostan	F	608.5 b*	0.92 hi
	M	539.4 de	0.94 gh
Markovo	F	390.6 i	1.32 a
	M	522.9 ef	1.07 cd
Bekleme	F	666.4 a	0.92 hi
	M	546.7 cd	1.07 c
*J. sibirica*			
Bekleme	F	562.7 c	0.99 f
	M	500.7 f	0.97 fg
Pirin	F	538 de	1.03 cde
	M	503.3 f	0.9 i
Vitosa	F	547.4 cd	1.05 cd
	M	505.5 f	1.03 de
*J. pygmaea*			
Dobrostan	F	466.4 g	1.11 b
	M	508.4 f	0.97 fg
Kamenlivitsa	F	439 h	1 ef
	M	435 h	1.13 b
Mursalitsa	F	549.6 cd	1.05 cd
	M	465.3 g	1.04 cde

M—male; F—female. * Arithmetic means marked with the same letters have no statistically proven differences.

**Table 4:** Duncan’s test of arithmetic means with respect to leaf anatomy at the species level.

Indicator	*J. communis*	*J. sibirica*	*J. pygmaea*
LH	545.7 a*	526.3 b	477.3 c
CC	1.04 a	0.99 b	1.05 a
TUCT	4.5 b	4.5 b	7.1 a
TLCT	4.7 b	5.1 a	4.5 c
THUS	4.5 a	3.1 c	3.7 b
THLS	5.4 a	3.8 c	4.3 b
THLA	10.9 a	8.8 b	9.1 b
TPP	91.2 b	95.2 a	87.4 b
TSP	224.3 a	186.2 b	214.5 a
WMV	435.6 a	404.4 b	407.4 b
HMV	157.1 a	131.4 b	159.8 a
WASC	152.4 a	141.6 b	116.9 c
HASC	167.2 a	146.7 b	126.6 c
SN	1529.7 a	1291.3 b	1005.7 c
SW	5.9a	5.79 b	5.4 c
SL	9.8 a	9.4 b	9.1 c

* Arithmetic means marked with the same letters have no statistically proven differences.

## Data Availability

The datasets generated during the current study are available from the corresponding author on reasonable request.

## Supplementary Materials

The following supporting information can be downloaded at https://www.mdpi.com/article/10.3390/plants13172419/s1, Table S1: Populations, coordinates, and meters above sea level (masl) of *Juniperus communis*, *J. sibirica*, and *J. pygmaea* collected from Bulgaria; Table S2: Abbreviations of the analyzed anatomical parameters in µm for *J. communis*, *J. sibirica*, and *J. pygmaea*; Figure S1: Abbreviations of the analyzed anatomical parameters for the leaves of the three species. Figure S2: Map of the collected samples of the three studied species in Bulgaria.
