# Supplementary material for: A Comparative Morphological and Anatomical Study of Juniperus communis L., J. sibirica Burgsd., and J. pygmaea K. Koch from Bulgaria
Source: Plants (Basel). 2024 Aug 29;13(17):2419. doi: 10.3390/plants13172419 (PMC11396953; doi:10.3390/plants13172419)
Supplement: Supplementary file 1 [file plants-13-02419-s001.zip › plants-3164517-supplementary.pdf]

Table S1. Populations, coordinates, and meters above sea level (masl) of *Juniperus communis*, *J. sibirica* and *J. pygmaea* collected from Bulgaria

	**Population**	**GPS co-ordi-nates; masl**	**Population**	**GPS co-ordi-nates; masl**	**Population**	**GPS co-ordi-nates; masl**
J.communiss	Bekleme	N42743 0.6 E 24617.1 1 1400 m asl	Dobrostan	N41541 2.3 E245502 .2 1308m asl	Markovo	N 420231 E244206 .4 529 m asl
J.sibirica	Vitosa	N42581 6.4 E23297. 26	Bekleme	N42907 2.5 E247136 .2 1500 мasl	Pirin	N41.753 811° E23.407 501° 2410ma sl
J. pygmaea	Kamenlivitsa	N42413 5.4 E245712 .2 1183 m asl	Dobrostan	N4154 12.3 E2455 02.2 1308 m asl	Mursalitsa	N41392 7.5 E2427 26.8 1628m asl

Table S2. Abbreviations of analyzed anatomical parameters in μm of *J. communis, J. sibirica, and J. pygmaea*

**Anatomical parameters in μm.**	**Abbreviations**
Leaf height	LH
Thickness of upper cover tissue	TUCT
Thickness of lower cover tissue	TLCT
Thickness hypodermis upper surface	THUS
Thickness hypodermis lower surface	THLS
Thickness hypodermis leaf angles	THLA
Thickness palisade parenchyma	TPP
Thickness spongy parenchyma	TSP
Width median vein	WMW
Height median vein	HMV
Width of the aperture of the secretory canal	WASC
Height of the aperture of the secretory canal	HASC
Stomata number	SN
Stomata width	SW
Stomata length	SL

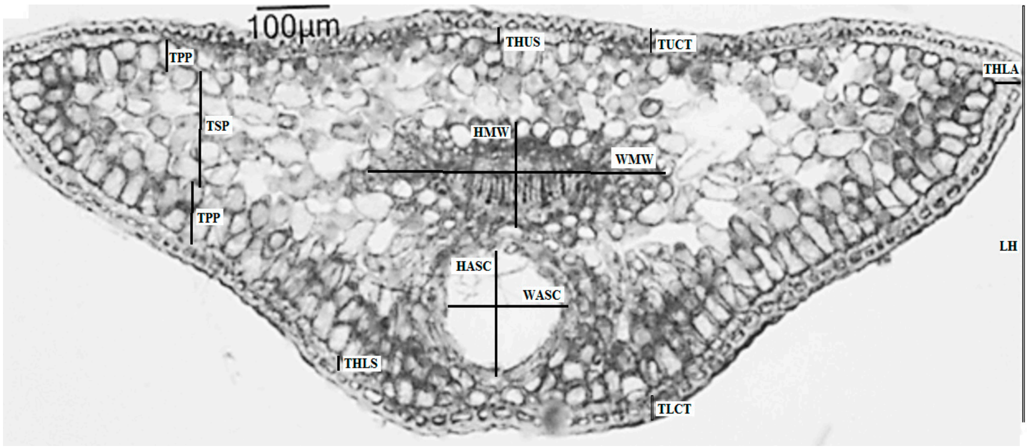

Figure S1. Abbreviations of analyzed anatomical parameters in the leaves of the three species.

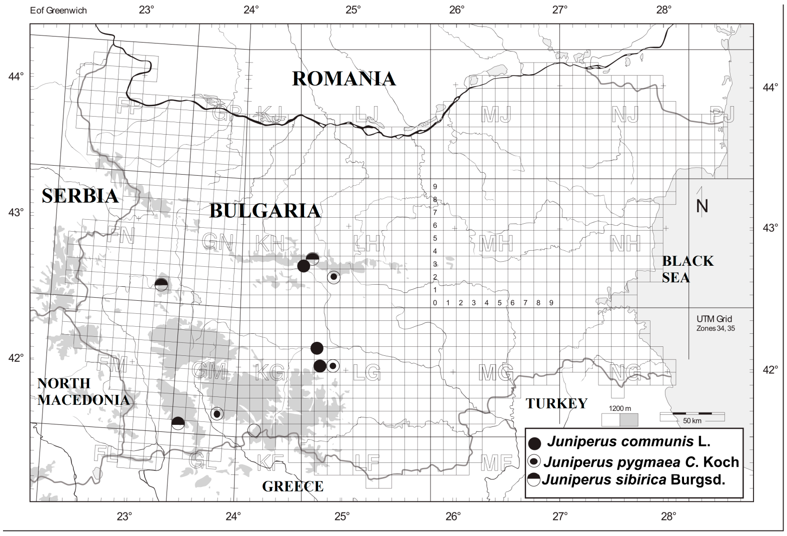

Figure S2. Map of the collected samples of the three studied species in Bulgaria**.**
